# Content and strength of conflict of interest policies at Scandinavian medical schools: a cross sectional study

**DOI:** 10.1186/s12909-022-03881-y

**Published:** 2022-11-26

**Authors:** Alice Fabbri, Shai Mulinari, Martin Johansson, Weda Ghaur, Abdullah Muhammad Khalil, Andreas Lundh

**Affiliations:** 1 Department for Health, University of Bath, Claverton Down, BA2 7AY Bath, UK; 2 Centre for Evidence-Based Medicine Odense (CEBMO), University of Southern Denmark and Odense University Hospital, Odense, Denmark; 3 Department of Sociology, Lund University, Lund, Sweden; 4 Department of Clinical Sciences Lund, Clinical Physiology, Lund University, Skåne University Hospital, Lund, Sweden; 5 Department of Paediatric Anaesthesia and Intensive Care, Children’s Hospital, Skane University Hospital, Lund University, Lund, Sweden; 6 Aarhus University, Århus, Denmark; 7 Medical University of Sofia, Sofia, Bulgaria; 8 Centre for Evidence-Based Medicine Odense (CEBMO) and Cochrane Denmark, Department of Clinical Research, University of Southern Denmark, Odense, Denmark; 9 Open Patient data Explorative Network (OPEN), Odense University Hospital, Odense, Denmark; 10 Department of Respiratory Medicine and Infectious Diseases, Bispebjerg Hospital, Copenhagen, Denmark

**Keywords:** Conflict of interest, Policies, Industry, Medical school, University

## Abstract

**Background:**

Concerns around staffs’ and students’ interactions with commercial entities, for example drug companies, have led several North American medical schools to implement conflict of interest (COI) policies. However, little is known about COI policies at European medical schools. We analysed the content and strength of COI policies at Scandinavian medical schools.

**Methods:**

We searched the websites of medical schools in Denmark, Norway, and Sweden and emailed the Deans for additional information. Using comparable methodology to previous studies, the strength of the COI policies was rated on a scale from 0 to 2 across 11 items (higher score more restrictive); we also assessed the presence of oversight mechanisms and sanctions.

**Results:**

We identified 77 unique policies for 15 medical schools (range 2–8 per school). Most of the policies (*n* = 72; 94%) were University wide and only five (6%) were specific for the medical schools. For six of eleven items one or more schools had a restrictive policy (score of two). None of the schools had a restrictive policy for the five additional items (speaking relationships, sales representatives, on-site education activities, medical school curriculum, and drug samples). Honoraria was the item with the highest score, with eight of the 15 schools having a score of two. Thirteen of the 15 schools had policies that identified a party responsible for policy oversight and mentioned sanctions for non-compliance.

**Conclusion:**

Our study provides the first evaluation of all Scandinavian medical schools’ COI policies. We found that the content of COI policies varies widely and still has shortcomings. We encourage Scandinavian medical schools to develop more stringent COI policies to regulate industry interactions with both faculty and students.

**Supplementary Information:**

The online version contains supplementary material available at 10.1186/s12909-022-03881-y.

## Background

The formative years of medical school play an important role in shaping future professional behaviour and critical thinking skills. One challenge for the quality of medical education is the influence of the drug and medical device industries. Educational materials, such as textbooks, may be influenced by commercial sources, and faculty may have financial ties that could influence clinical practice, teaching and training [1–4]. Medical schools therefore have an important role to play to ensure that their students are trained about professional-industry interactions and are protected from commercial influences during their training.

Concerns around interactions with commercial entities have led some medical schools to implement specific conflict of interest (COI) policies [5]. Such policies have been shown to affect both attitudes and behaviours of students [6, 7]. For example, a study found that physicians who had been exposed to COI policies during their residency training less frequently prescribed heavily marketed antidepressants [6].

In 2007, the American Medical Student Association (AMSA) developed a scorecard and started annual assessments of COI policies across US medical schools [8]. This was instrumental in raising awareness on COI among academic institutions; for example, the media attention generated by the AMSA scorecard influenced the development of COI policies in several US medical schools [8] and the institutional scores have improved year after year likely due to these rankings. Moreover, the AMSA scorecard has since been adapted and used in similar studies conducted in Australia, Belgium, Canada, France, and Germany [9–13].

However, the majority of the studies were conducted in North America and little is known about COI policies at European medical schools apart from the three studies from Central Europe [9, 11, 13]. We therefore decided to investigate the content and strength of COI policies at Scandinavian medical schools.

## Methods

Our study was based on a protocol (Supplementary File 1) using methodology similar to previous studies on COI policies at medical schools in US and Canada [8, 12]. The protocol specifies the methods for the identification, assessment, and analysis of COI policies at Scandinavian medical schools.

### Identification of conflict of interest policies

Two pairs of coders (one pair for Sweden and one pair for both Denmark and Norway) developed a list of the medical schools in the three included countries using Google searches. The names and number of medical schools per country was further verified by searching official national educational websites. (see Supplementary File 2) We included Scandinavian medical schools with full Bachelor and Master’s program allowing graduates to work as physicians after completing the program. Schools where the majority of the program took place in another country were excluded. Two pairs of coders independently searched the website of each included school and its parent University in July-August 2020 to identify COI policies. Disagreements were resolved by discussion. We use the term medical school to describe the institution hosting the medical education, typically a faculty of health sciences.

The websites were searched using a list of keywords in appropriate language (e.g., *policy, conflict of interest, industry*) and was complemented with Google searches (see Supplementary File 3 for the full details of the search strategy). The name of each policy and the date of adoption or most recent amendment were recorded. Both policies of the medical school and University-wide policies were included in line with the previous study by Shnier et al. [12]. In some cases, the relevant information was only stated on a website and not contained in a separate document. In those cases, we included the website information as a form of informal policy.

A letter in the appropriate language was sent to the Dean of each medical school to inform them of the study. The letter explained the aim of the study, listed the COI policies identified via the website searches and asked for confirmation that we had not missed any relevant documents. We asked for both publicly available and non-publicly available policies. The first email was sent in September 2020 and was followed by up to two e-mail reminders in case the Dean did not reply. In only one case we did not receive a reply nor a delivery notification that confirmed the receipt of the email after two e-mail reminders. We therefore undertook additional contact by multiple telephone calls and one additional email, but were unable to determine whether the Dean had received our email.

We did not include specific COI policies of the various teaching hospitals affiliated with a particular medical school as these institutions are typically not under the authority of the medical school (i.e., faculty of health sciences). Similarly, we did not include regional or national policies or regulation (even when Deans sent us copies of those) unless they were explicitly mentioned in the included COI policies or on the websites of the University or medical school. When a national or external policy was mentioned in an included COI policy or on the medical school or University websites, it was used for the assessment, but it did not contribute to the final count of institutional policies in order to avoid double-counting.

### Assessment of content and strength of conflict of interest policies

We based our assessment of the content and strength of COI policies on the 12-item adaptation of the AMSA scorecard developed by Shnier et al. for medical schools in Canada [12]. We modified the system slightly to adapt it to the Scandinavian context. For example, we excluded the item on “Industry support for scholarships and funds for trainees” because industry does not provide scholarships to medical students in Scandinavian countries as education is free for EU/EEA students.

Our revised 11-item assessment system included the following items:

1. gifts (including meals)
2. consulting relationships (excluding funding for scientific research and speaking fees)
3. industry-funded speaking relationships and speakers’ bureaus
4. honoraria (beyond consulting or speaker fees)
5. ghostwriting
6. disclosure of financial relationships with industry
7. industry sales representatives
8. on-site education activities
9. compensation for travel or attendance at off-site lectures and meetings
10. medical school curriculum (or other documentation of educational objectives aimed at training students in industry-interactions. In order to assess this item, we looked not only at the COI policies but also at the medical curriculum and learning objectives of the school of medicine. However, those documents were not included in our count of the number of institutional COI policies for each school)
11. drug samples

Each item was graded using a rating scale of 0 to 2 (0 = no policy or permissive policy, 1 = moderate, and 2 = restrictive policy). The system also included two final questions on oversight and sanction that were graded as “Yes” or “No”.

We developed a standardised guidance on how to assess the different items. Supplementary File 4 shows the detailed assessment criteria for each item. We pilot tested the system using three randomly selected Canadian medical schools included in the study by Shnier et al. [12]. This allowed training of the data collectors and to address ambiguities identified through coding disagreements.

Two pairs of coders (one pair for Sweden and one pair for both Denmark and Norway) assessed the included COI policies of each medical school independently. Disagreements were resolved by discussion. If consensus could not be reached, a third assessor adjudicated.

### Data analysis

For each included country, we reported scores for the 11 policy items and for oversight and sanctions. We also reported the mean item score per country.

### Ethical issues

Since the study focused on institutional policies, rather than personal conflicts of interest information, no ethics approval was required according to Scandinavian law.

## Results

We identified four medical schools in Denmark, five in Norway and seven in Sweden. One Norwegian medical school was excluded, as the major part of the education was done in Hungary, which led to inclusion of four Norwegian medical schools. (see Supplementary File 2) Fourteen of 15 schools responded to our email request.

Combining website searches and email responses, we identified a total of 77 unique institutional policies: 13 from Denmark, 19 from Norway, and 45 from Sweden (median 6; range 2–8 policies per school). (Supplementary File 5) Most of the policies (*n* = 72; 94%) were University wide and only five (6%) were specific for the medical schools. Most of the policies (65%, *n* = 50) were adopted or reviewed between 2016 and 2020.

Table 1 summarises the number of medical schools with policies in each category and the strength of the policy for the three included countries. For six of eleven items one or more schools had a restrictive policy (i.e., score of 2). However, no school had a restrictive policy for the five additional items (speaking relationships, sales representatives, on-site education activities, medical school curriculum, and drug samples). The item with the most frequent number of restrictive policies was honoraria (8 of 15 schools had a score of 2). Thirteen of the 15 schools had policies that identified a party responsible for the oversight of the policy and mentioned sanctions for non-compliance.

**Table 1:** Number of medical schools with policies for each item and strength of the policy

Country	DENMARK (*n* = 4)			NORWAY (*n* = 4)			SWEDEN (*n* = 7)			TOTAL (*n* = 15)		
**Items**	No. of schools with no or permissive policy (score = 0)	No. of schools with moderate policy (score = 1)	No. of schools with restrictive policy (score = 2)	No. of schools with no or permissive policy (score = 0)	No. of schools with moderate policy (score = 1)	No. of schools with restrictive policy (score = 2)	No. of schools with no or permissive policy (score = 0)	No. of schools with moderate policy (score = 1)	No. of schools with restrictive policy (score = 2)	No. of schools with no or permissive policy (score = 0)	No. of schools with moderate policy (score = 1)	No. of schools with restrictive policy (score = 2)
1.Gifts	1	3	0	0	3	1	0	7	0	1	13	1
2.Consulting	1	0	3	0	2	2	0	7	0	1	9	5
3.Speaking	4	0	0	2	2	0	0	7	0	6	9	0
4.Honoraria	3	1	0	0	2	2	0	1	6	3	4	8
5.Ghostwriting	2	0	2	3	1	0	4	1	2	9	2	4
6.Disclosure	0	4	0	0	2	2	0	7	0	0	13	2
7.Sales representatives	4	0	0	2	2	0	7	0	0	13	2	0
8.On-site education	3	1	0	2	2	0	5	2	0	10	5	0
9.Travel or off-site events	2	1	1	1	0	3	0	7	0	3	8	4
10.Medical school curriculum	4	0	0	2	2	0	7	0	0	13	2	0
11.Samples	4	0	0	3	1	0	6	1	0	13	2	0
Mean item score^a^	0.5			0.9			0.7			0.7		
**Enforcement of policies**												
	**No**	**Yes**		**No**	**Yes**		**No**	**Yes**		**No**	**Yes**	
Oversight	1	3		1	3		0	7		2	13	
Sanctions	1	3		1	3		0	7		2	13	

^a^Calculated as the sum of the scores divided by number of items (i.e., 11) and number of institutions

On average, Norwegian schools had stricter policies (mean score: 0.9) followed by Sweden (mean score: 0.7) and Denmark (mean score: 0.5).

## Discussion

Our study provides the first evaluation of Scandinavian medical schools’ COI policies. The policies were primarily University-wide and although we found relevant policies for all the included institutions, the number and content of policies varied widely and there are still substantial shortcomings. Lack of restrictive policies were often related to issues unique to medicine. For example, none of the included schools had a restrictive policy for contacts with sales representatives and drug samples.

### Strengths and limitations

A key strength of our study is that it is based on a robust methodology that has been previously used in studies from North America and Central Europe. We used multiple methods (i.e., website search and contacts with Deans) to retrieve relevant institutional policies and each policy was independently assessed by two coders. The study also has some limitations. First, we might have missed relevant COI policies as we used only specific keywords and policies may have been indexed under other terms or may not be publicly available. Moreover, the search engines can work differently on different Universities’ websites. However, we managed to get replies about relevant documents from 14 of 15 institutions. Second, we focused on COI policies at medical school or University-wide level and did not include policies of teaching hospitals or regional or national regulations unless they were explicitly mentioned in the included COI policies or on the medical school or University websites. This means our study provides only a partial picture of the regulation in place in each of the included countries. Third, the system we used for our assessment has some limitations. For example, it does not address COI in research or how academic institutions deal with the risk of industry funders’ influence on study design, analysis, and reporting. Fourth, the results of this study might underestimate the teaching activities on COI as our assessment of that item was mainly based on the learning objectives listed in the official medical curricula and not an examination of the teaching materials or content of lectures. Fifth, we used the same coding system for the assessment of institutional policies from the three countries to allow for a cross country comparison. Not tailoring the assessment to each national system could potentially reduce granularity. However, we believe that the Scandinavian healthcare and education system is fairly similar to allow for this strategy. Finally, while most Universities and schools had mechanisms in place for oversight and sanctions for non-compliance, we did not assess the extent of enforcement.

### Context and perspective

Similar studies of medical schools in France and Germany found weak content of COI policies [9, 11]. However, the results of the other European studies are not directly comparable to ours as those studies only focused on medical schools’ policies while we also included policies of the parent University (which represented the majority of the policies in our sample). The content of COI policies at Scandinavian medical schools would be weaker had we excluded University-wide policies. The lack of medical school specific policies is somewhat striking since several domains addressed by our assessment system are unique for medicine (e.g., drug samples and drug representatives) and will not be covered by University-wide policies suited for all faculties.

However, as previously mentioned in the limitation section, we did not include policies of teaching hospitals or regional or national regulation unless they were explicitly mentioned in the included COI policies. We made this decision as we were interested in the guidance that University staff or students can find on the website or in the official documents of their institution. This means our study provides only a partial picture of the actual situation in Scandinavia. For example, clinically active lecturers at Scandinavian medical schools are almost always under a dual employment, both by the University and the associated hospital which is run by the local authority. Similarly, medical students conducting clinical rotations at University associated hospitals are obliged by hospital protocol to follow these same rules and regulations. A future study could investigate COI policies at Scandinavian teaching hospitals as they play an important role in training of healthcare professionals. Furthermore, some relationships with industry are also regulated by national laws or guidelines. For example, in Denmark, the distribution of drug samples is regulated by both a national law [14] and the ethical guidelines of the Danish Pharmaceutical Industry and the Danish Medical Association [15, 16]. In Sweden, university staff are considered civil servants and should follow legal provisions governing “secondary employments” (e.g. consultancies), and gifts and procurement due to risk of bribing [17]. Furthermore, since 2015 the ethical guidelines of the Swedish Pharmaceutical Industry prohibits companies from paying for healthcare professionals travel or accommodation at conferences and meetings [18]. In Norway, according to the Health Personnel Act, healthcare professionals must not receive gifts or other benefits that could unduly influence their actions [19]. Additional ethical guidance on relationships with the pharmaceutical industry is also provided by professional associations like the Norwegian Medical Association [20].

These examples illustrate the challenges with comparing content of COI policies between different nations. Our findings of shortcomings in the official COI policies of Scandinavian medical schools do not mean that the situation in Scandinavia is problematic since the industry is also regulated at both hospital and national levels. Furthermore, the relationships between healthcare professionals and the industry are also managed by the ethical guidelines of the Scandinavian medical associations and the pharmaceutical industry. However, it is worth mentioning that these voluntary guidelines are sometimes less restrictive compared to legally-binding regulation [21]. In light of this, we believe it is important that University policies also address the issue either by having strict policies or clearly referring to applicable regulation and industry guidelines so that staff have a single site to go to instead of needing to know about multiple national or regional laws, regulations, and guidelines.

Finally, it is important to note that the presence of an institutional policy does not necessarily mean that all its provisions are accurately implemented, though, it could still provide an important roadmap for staff and students. The formative years of medical school play an important role in shaping future professional behaviour and there is also evidence showing that being trained in an institution with a strict COI policy may lead to more rational drug prescribing behaviours [22]. We encourage Scandinavian medical schools to develop and implement more restrictive COI policies in the interest of both patients and society. Our study provides a comprehensive catalogue of identified policies, which institutions may use as a source of inspiration to revise their current policies.

## Supplementary Information

**Additional file 1: Supplementary File 1.** Protocol.  Conflict of interest policies at Danish,Swedish, and Norwegian medical schools.

**Additional file 2: Supplementary File 2. **List of Scandinavian medical schools/universities.

**Additional file 3: Supplementary File 3.** Methods to identify the conflict of interest policies.

**Additional file 4: Supplementary File 4.** Assessment system for the institutional conflict of interest policies.

**Additional file 5: Supplementary File 5.** List of included policies.

## Data Availability

The data used and/or analysed for the current study, including scores at institutional level, are available from the corresponding author on reasonable request.
