# Correction: Content and strength of conflict of interest policies at scandinavian medical schools: a cross sectional study

**DOI:** 10.1186/s12909-023-04128-0

**Published:** 2023-03-02

**Authors:** Alice Fabbri, Shai Mulinari, Martin Johansson, Weda Ghaur, Abdullah Muhammad Khalil, Andreas Lundh

**Affiliations:** 1 Department for Health, University of Bath, Claverton Down, Bath, BA2 7AY UK; 2 Centre for Evidence-Based Medicine Odense (CEBMO), University of Southern Denmark and Odense University Hospital, Odense, Denmark; 3 Department of Sociology, Lund University, Lund, Sweden; 4 Department of Clinical Sciences Lund, Clinical Physiology, Skåne University Hospital, Lund University, Lund, Sweden; 5 Department of Paediatric Anaesthesia and Intensive Care, Children’s Hospital, Skane University Hospital, Lund University, Lund, Sweden; 6 Aarhus University, Århus, Denmark; 7 Medical University of Sofia, Sofia, Bulgaria; 8 Centre for Evidence-Based Medicine Odense (CEBMO) and Cochrane Denmark, Department of Clinical Research, University of Southern Denmark, Odense, Denmark; 9 Open Patient data Explorative Network (OPEN), Odense University Hospital, Odense, Denmark; 10 Department of Respiratory Medicine and Infectious Diseases, Bispebjerg Hospital, Copenhagen, Denmark

**Correction To: BMC Medical Education (2022) 22:812**

10.1186/s12909-022-03881-y.

Following publication of the original article [1], the authors informed us that due to an error while merging the data, Table 1 includes some inaccurate data. Please find below the amended version of the table. To reflect these changes, one sentence of the results section should be amended.

Previous version: *“On average, Norwegian schools had stricter policies (mean score: 0.9) followed by Sweden (mean score: 0.7) and Denmark (mean score: 0.5)”.*

Amended version: *“On average, Norwegian schools had stricter policies (mean score: 0.8) followed by Sweden (mean score: 0.7) and Denmark (mean score: 0.5)”.*

**Table 1:** Number of medical schools with policies for each item and strength of the policy

Country	DENMARK (n = 4)			NORWAY (n = 4)			SWEDEN (n = 7)			TOTAL (n = 15)		
**Items**	No. of schools with no or permissive policy (score = 0)	No. of schools with moderate policy (score = 1)	No. of schools with restrictive policy (score = 2)	No. of schools with no or permissive policy (score = 0)	No. of schools with moderate policy (score = 1)	No. of schools with restrictive policy (score = 2)	No. of schools with no or permissive policy (score = 0)	No. of schools with moderate policy (score = 1)	No. of schools with restrictive policy (score = 2)	No. of schools with no or permissive policy (score = 0)	No. of schools with moderate policy (score = 1)	No. of schools with restrictive policy (score = 2)
1.Gifts	1	3	0	0	3	1	0	7	0	1	13	1
2.Consulting	1	0	3	0	2	2	0	7	0	1	9	5
3.Speaking	4	0	0	2	2	0	0	7	0	6	9	0
4.Honoraria	3	1	0	0	2	2	0	1	6	3	4	8
5.Ghostwriting	2	0	2	3	1	0	4	1	2	9	2	4
6.Disclosure	0	4	0	0	2	2	0	7	0	0	13	2
7.Sales representatives	4	0	0	2	2	0	7	0	0	13	2	0
8.On-site education	3	1	0	3	1	0	5	2	0	11	4	0
9.Travel or off-site events	2	1	1	1	0	3	0	7	0	3	8	4
10.Medical school curriculum	4	0	0	3	1	0	6	1	0	13	2	0
11.Samples	4	0	0	3	1	0	7	0	0	14	1	0
Mean item score^*^	0.5			0.8			0.7			0.7		
**Enforcement of policies**												
	**No**	**Yes**		**No**	**Yes**		**No**	**Yes**		**No**	**Yes**	
Oversight	1	3		1	3		0	7		2	13	
Sanctions	1	3		1	3		0	7		2	13	

* Calculated as the sum of the scores divided by number of items (i.e., 11) and number of institutions.
