# Supplementary material for: Content and strength of conflict of interest policies at Scandinavian medical schools: a cross sectional study
Source: BMC Med Educ. 2022 Nov 26;22:812. doi: 10.1186/s12909-022-03881-y (PMC9701355; doi:10.1186/s12909-022-03881-y)
Supplement: Supplementary file 1 — Additional file 1: Supplementary File 1. Protocol. Conflict of interest policies at Danish,Swedish, and Norwegian medical schools. [file 12909_2022_3881_MOESM1_ESM.docx]

**Supplementary File 1.**

**Protocol**

**Conflict of interest policies at Danish, Swedish, and Norwegian medical schools**

(Final version: July 2020)

**Background and Objectives**

Commercial actors such as the drug and device industry may influence medical education through their impact on biomedical research, educational content, and professional behaviours.(1) Schools of Medicine therefore have a role in ensuring that their students are trained about professional-industry interactions and are protected from undue commercial influence during their training. Since 2007, the American Medical Student Association (AMSA) has published an annual scorecard ranking American medical schools on their conflict of interest (COI) policies.(2) The AMSA scorecard has been adapted and used in similar studies that have been conducted in Canada, France, and Germany.(3-5) We are planning to conduct a similar study in Denmark, Sweden, and Norway.

**Methods**

***Identification of conflicts of interest policies***

One pair of coders for each country will develop a list of the medical schools in Denmark, Norway, and Sweden and independently search the website of each school and its parent University to identify COI policies. Disagreements will be resolved by discussion.

The websites will be searched using a list of keywords in appropriate language (e.g., *policy, conflict of interest, industry*) and will be complemented with google searches. Both policies of the medical school and University-wide policies will be included.

We will not include specific COI policies of the various teaching hospitals affiliated with a particular medical school as these institutions are typically not under the authority of the medical school (i.e., faculty of health sciences). Similarly, we will not include regional or national policies or regulation unless they are explicitly mentioned in the included COI policies or on the websites of the University or medical school. When a national or external policy is mentioned in an included COI policy or on the medical school or University websites, it will be used for the assessment, but it will not contribute to the final count of institutional policies.

A letter in the appropriate language will be sent to each Dean of the medical school to inform them of the study and ask for confirmation that we have not missed any relevant documents. The first email will be followed by up to two e-mail reminders in case the Deans do not reply.

***Assessment of content and strength of conflicts of interest policies***

For assessment of the content and strength of COI policies, we will use an adaptation of the AMSA scorecard developed by Shnier et al for medical schools in Canada.(5) We will modify the scorecard slightly to adapt it to the Scandinavian context. Each item will be graded using a rating scale of 0 to 2 (0 = no policy or permissive policy, 1 = moderate, and 2 = restrictive policy). We will develop a standardised guidance on how to assess the different items and pilot test the scorecard. This will allow training of the data collectors.

One pair of coders for each country will assess the included COI policies of each medical schools independently. Disagreements will be resolved by discussion. If consensus cannot be reached, a third assessor will adjudicate.

***Data analysis***

For each included country, we will report scores for all the policy items.

**References**

1. Institute of Medicine (US) Committee on Conflict of Interest in Medical Research E, and Practice; Lo B, Field MJ, editors. Conflict of Interest in Medical Research, Education, and Practice. National Academies Press (US); 2009.

2. Carlat DJ, Fagrelius T, Ramachandran R, Ross JS, Bergh S. The updated AMSA scorecard of conflict-of-interest policies: a survey of U.S. medical schools. BMC Medical Education. 2016;16(1):202.

3. Grabitz P, Friedmann Z, Gepp S, Hess L, Specht L, Struck M, et al. Quantity and quality of conflict of interest policies at German medical schools: a cross-sectional study and survey. 2020;10(9):e039782.

4. Scheffer P, Guy-Coichard C, Outh-Gauer D, Calet-Froissart Z, Boursier M, Mintzes B, et al. Conflict of interest policies at French medical schools: Starting from the bottom. PLoS ONE. 2017;12 (1).

5. Shnier A, Lexchin J, Mintzes B, Jutel A, Holloway K. Too Few, Too Weak: Conflict of Interest Policies at Canadian Medical Schools. PLoS ONE. 2013;8 (7).
