# Supplementary material for: Content and strength of conflict of interest policies at Scandinavian medical schools: a cross sectional study
Source: BMC Med Educ. 2022 Nov 26;22:812. doi: 10.1186/s12909-022-03881-y (PMC9701355; doi:10.1186/s12909-022-03881-y)
Supplement: Supplementary file 2 — Additional file 2: Supplementary File 2. List of Scandinavian medical schools/universities. [file 12909_2022_3881_MOESM2_ESM.doc]

**Supplementary File 2 : List of Scandinavian medical schools/universities**

Identification of the medical schools: four co-authors of this study (WG, AMK, MJ, AL) are either studying Medicine or working as physicians in Scandinavian countries and are therefore familiar with the medical education system in the included countries. In addition to the Google searches the name and number of medical schools per country was verified by searching official national educational websites (See Table S2).

**Table S1. List of the Scandinavian medical schools/universities and the Internet sites used for the web research**

| **University** | **Website** |
| --- | --- |
| **DENMARK** | |
| Aarhus University | Medical School: https://health.au.dk/en/  University: https://www.au.dk |
| University of Copenhagen | Medical School: https://sund.ku.dk  University: https://www.ku.dk/ |
| University of Southern Denmark | Medical School: https://www.sdu.dk/da/om_sdu/fakulteterne/sundhedsvidenskab  University:  https://www.sdu.dk/ |
| Aalborg University | Medical School: https://www.sundhedsvidenskab.aau.dk  University: https://www.aau.dk |

| **University** | **Website** |
| --- | --- |
| **NORWAY** | |
| University of Oslo | Medical School: https://www.med.uio.no  University: https://www.uio.no/ |
| Norwegian University of Science and Technology | Medical School: https://www.ntnu.edu/mh/faculty-of-medicine-and-health-sciences  University: https://www.ntnu.edu |
| Tromsø University | Medical School: https://en.uit.no/enhet/helsefak  University: https://uit.no/ |
| Bergen University | Medical School: https://www.uib.no/en/med  University: https://www.uib.no |

| **University** | **Website** |
| --- | --- |
| **SWEDEN** | |
| Stockholm | Medical School: https://ki.se/  University: https://ki.se/ |
| Gothenburg | Medical School: https://www.gu.se/sahlgrenska-akademin  University: https://www.gu.se |
| Umea | Medical School: https://www.umu.se/medicinsk-fakultet/  University: https://www.umu.se/ |
| Linköping | Medical School: https://liu.se/organisation/liu/medfak  University: https://liu.se/ |
| Lund | Medical School: https://www.medicin.lu.se/  University: https://www.lu.se/ |
| Uppsala | Medical School: https://www.medsci.uu.se/  University: https://www.uu.se/ |
| Örebro | Medical School: https://www.oru.se/institutioner/medicinska-vetenskaper/  University: https://www.oru.se/ |

**Table S2. Websites used to verify the list of included Medical Schools.**

| **Denmark** | We identified and included four medical schools. The following official educational guidance website lists the four medical schools we included: [https://www.ug.dk/uddannelser/bachelorogkandidatuddannelser/bacheloruddannelser/sundhedsvidenskabeligebacheloruddannelser/medicin](https://eur01.safelinks.protection.outlook.com/?url=https%3A%2F%2Fwww.ug.dk%2Fuddannelser%2Fbachelorogkandidatuddannelser%2Fbacheloruddannelser%2Fsundhedsvidenskabeligebacheloruddannelser%2Fmedicin&data=05|01|af987@bath.ac.uk|7163863b471f42b0b2b508daa917b2e1|377e3d224ea1422db0ad8fcc89406b9e|0|0|638008216494256379|Unknown|TWFpbGZsb3d8eyJWIjoiMC4wLjAwMDAiLCJQIjoiV2luMzIiLCJBTiI6Ik1haWwiLCJXVCI6Mn0%3D|3000|||&sdata=OcIW%2F4xcMCe8IpN08iggD6NLEe%2FeEFYBLaxVQZJ7BBU%3D&reserved=0) |
| --- | --- |
| **Norway** | We identified five medical schools and included four. We identified a medical school program where students take the first year at Oslo New University College and the next five years in Hungary. We decided not to include it as the program is mostly based in Hungary. The following official educational guidance website lists the Norwegian medical schools: [https://utdanning.no/studiebeskrivelse/medisin_profesjonsstudium](https://eur01.safelinks.protection.outlook.com/?url=https%3A%2F%2Futdanning.no%2Fstudiebeskrivelse%2Fmedisin_profesjonsstudium&data=05|01|af987@bath.ac.uk|f000fb0cd9db450d57ee08daac3e352a|377e3d224ea1422db0ad8fcc89406b9e|0|0|638011680426814534|Unknown|TWFpbGZsb3d8eyJWIjoiMC4wLjAwMDAiLCJQIjoiV2luMzIiLCJBTiI6Ik1haWwiLCJXVCI6Mn0%3D|3000|||&sdata=htUehh8CE4B%2FJo5NHehR86xpobEPbDtT9d8p2VrDbIg%3D&reserved=0) |
| **Sweden** | We identified and included seven medical schools. The following websites list the seven medical schools we included:   1. Centralised Swedish application system used by all Universities and students:   <https://www.antagning.se/se/search?period=16&freeText=Läkarprogrammet>   1. Link to the official decision by the relevant state authority (Swedish Higher Education Authority) to grant the seven universities the right to have medical education: [https://www.uka.se/om-oss/aktuellt/nyheter/2020-11-06-sju-larosaten-far-tillstand-att-utfarda-ny-lakarexamen.html](https://eur01.safelinks.protection.outlook.com/?url=https%3A%2F%2Fwww.uka.se%2Fom-oss%2Faktuellt%2Fnyheter%2F2020-11-06-sju-larosaten-far-tillstand-att-utfarda-ny-lakarexamen.html&data=05|01|af987@bath.ac.uk|7163863b471f42b0b2b508daa917b2e1|377e3d224ea1422db0ad8fcc89406b9e|0|0|638008216494256379|Unknown|TWFpbGZsb3d8eyJWIjoiMC4wLjAwMDAiLCJQIjoiV2luMzIiLCJBTiI6Ik1haWwiLCJXVCI6Mn0%3D|3000|||&sdata=AX%2FhDQZNpZSPcRIjs4jCU%2FxAhd8zfKZ84FuooY70Hb8%3D&reserved=0) |
