# Supplementary material for: Content and strength of conflict of interest policies at Scandinavian medical schools: a cross sectional study
Source: BMC Med Educ. 2022 Nov 26;22:812. doi: 10.1186/s12909-022-03881-y (PMC9701355; doi:10.1186/s12909-022-03881-y)
Supplement: Supplementary file 3 — Additional file 3: Supplementary File 3. Methods to identify the conflict of interest policies. [file 12909_2022_3881_MOESM3_ESM.docx]

**Supplementary File 3. Methods to identify the conflicts of interest policies**

1. Search for the following **keywords in the search engine of the website**. Apart from the keyword search, spend some time searching the website (check multiple pages and links).
2. Use also **google search** (e.g., conflict of interest + name of the University you are assessing).

| **English** | **Danish** | **Norwegian** | **Swedish** |
| --- | --- | --- | --- |
| policy, policies | politik, politikker | politikk | policy, policies |
| guideline, regulation, ethics code | retningslinjer/vejledning,  regulering/styring/kontrol,  etisk kodeks/regler | retningslinjer,  regulering  etikk, etiske retningslinjer | riktlinje,  styrdokument,  reglering,  regulation,  etik |
| conflict of interest, conflicts of interest, COI, Competing interest | interessekonflikt/modstridende interesser,  konkurrerende interesser | interessekonflikt(er), konkurrerende interesse/virksomhet | intressekonflikter, konkurrerande verksamhet |
| industry,  industry interaction | industri/erhverv,  interaktion med industrien | industri,  industri interaksjon | industri,  interaktion med industrin |
