# Supplementary material for: Content and strength of conflict of interest policies at Scandinavian medical schools: a cross sectional study
Source: BMC Med Educ. 2022 Nov 26;22:812. doi: 10.1186/s12909-022-03881-y (PMC9701355; doi:10.1186/s12909-022-03881-y)
Supplement: Supplementary file 4 — Additional file 4: Supplementary File 4. Assessment system for the institutional conflict of interest policies. [file 12909_2022_3881_MOESM4_ESM.docx]

**Supplementary File 4. Assessment system for the institutional COI policies** (adapted from Shnier et al., Plos One 2013)

**1. Gifts (including meals)**

2 = All gifts and on-site meals funded by industry are prohibited, regardless of nature or value.

1 = Less stringent limitation on industry-funded gifts (e.g., gifts prohibited above a certain monetary threshold [no matter what the limit is]– or gifts prohibited but meals allowed).

0 = No policy, or policy that would not substantially reduce gifting (e.g., gifts are allowed but discouraged, or limited in a non-specific way to “appropriate,” or primarily for the benefit of patients).

**2. Consulting relationships (excluding scientific research and speaking)**

2 = Consulting relationships with industry must be subjected to institutional review or approval. Additionally, they must either be described in a formal contract, or payment for services must be commensurate to the task.

Note for the coders: by formal contract, we mean that the formal contract needs to be approved by the University. If the policy specifies an amount (a max. salary per hour), then it is considerate commensurate to the task.

1 = As above, without the institutional review or approval requirement.

0 = No policy, or policy that would allow consulting relationships to occur without institutional scrutiny or that would allow relationships in which payments are not commensurate with work.

**3. Industry-funded speaking relationships/speakers’ bureaus**

2 = Speaking relationships that may play a role in product marketing are not allowed, nor are excessive speaker fees that may function as de facto gifts. An effective policy must not implicitly permit (a) long-term speaking agreements or (b) industry to have a role in determining presentation content. (Some effective policies may explicitly prohibit participation in a speakers’ bureau. Other effective policies contain elements such as limits on compensation and reimbursement and a requirement to ensure the scientific integrity of information presented.)

 1 = Industry-funded speaking relationships are regulated, but with less stringent limits on longevity, content or compensation.

0 = No policy, or policy that does not define the limits on longevity, content or compensation.

**4. Honoraria**

By honoraria we mean any financial relationship apart from what was already specified in Categories 1, 2, and 3 above.

2 = No acceptance of honoraria; compensation must be at fair market value and publicly disclosed

1 = Limits on accepting/disclosing honoraria.

0 = No policy, or no limits on acceptance.

**5. Ghostwriting**

2 = Ghostwriting is not permitted.

1 = Few or no restrictions; management is left to individual discretion.

0 = No policy.

Note for the coders: institution needs to specifically mention the issue of ghostwriting/guest authorship (a phenomenon by which an industry representative/employees writes the paper, but is not recognised as an author in the final paper which is then signed by the academic researcher). If the institution’s policy only has a vague sentence that says that researchers should respect the ICMJE criteria for authorship (ICMJE indirectly prohibits ghost-authorship), this is not sufficient to give 2 points.

**6. Disclosure**

2 = Personnel are required to disclose past and present financial relationships with industry (e.g., consulting and speaking agreements, research grants) on a publicly available website

1 = Universally-required, internal disclosure to the medical school or hospital administration. (Policies requiring disclosure only when presenting or publishing do not meet this criterion.)

0 = No policy/No disclosure.

**7. Industry Sales Representatives**

2 = Pharmaceutical and device representatives are not allowed to meet with faculty regardless of location, or are not permitted to market their products anywhere inside the medical center and associated clinics and offices. (Exceptions may be made for non-marketing purposes, such as training on devices or equipment.)

Note for the coders: If the policies say medical students/doctors should not be subject to these interactions or say that sales reps are not allowed on campus or University ground, we will give 2 points.

 1 = Pharmaceutical representatives are permitted to meet with faculty, but with significant limitations (e.g., only in non-patient care areas or only by appointment). Exceptions as above.

 0 = No policy, or policy that does not substantially limit access.

**8. On-site Education Activities**

2 = Industry is not permitted to provide direct financial support for educational activities, including Continuing Medical Education (CME) activities for University staff/faculty, directly or through a subsidiary agency.  (However, companies may contribute unrestricted funds to a central fund or oversight body at the academic medical center, which, in turn, would pool and disburse funds for programs that are independent of any industry input or control.)

1 = Less stringent limitations to ensure independence of educational content (e.g., standards to establish freedom from industry influence of content, such as review and approval of presentations; language that prevents industry from selecting the speaker; or language such as: industry funding may be allocated for a particular topic, but must be provided directly to the department, not to individuals).

0 = No policy, or a policy that would not substantially limit industry influence over educational activities (e.g., industry funding must be disclosed).

**9. Compensation for Travel or Attendance at Off-site Lectures & Meetings**

2 = Personnel may not accept payment, gifts or financial support from industry to attend lectures and meetings. (An exception may be made for modest meals, if part of a larger program.) Travel support may only be accepted if it is subject to institutional approval from the University or industry is prevented from selecting (“earmarking”) the recipients. Note: speaking and consulting relationships are evaluated separately in domain 1.

1 = Less stringent limitations.

0 = No policy, or a policy that would not substantially limit participation in industry-funded events and meetings.

**10. Medical school curriculum (or other documentation of educational objectives/course content)**

Note for coders: in order to answer this question, first have a look at the COI policy.

Then go to the Medical curriculum and look at the learning objectives of the School of Medicine; however, these documents will not be included in the count of the number of institutional COI policies for each school.

2 = Students are trained to understand institutional conflict-of-interest policies and recognize how industry promotion can influence clinical judgment. (e.g., the policy or the learning objectives suggest there should be courses/teaching on both the COI policies and on conflict of interest)

1 = Curriculum addresses conflict of interest in a more limited way (e.g., the learning objectives mention training on the COI policy only or training on conflict of interest only.)

0 = No policy (not addressed in curriculum or elsewhere).

**11. Samples**

2 = Industry samples are prohibited.

1 = Samples or vouchers for medications may be provided, but with significant limitations (e.g., samples may not be given directly to physicians, samples must be dispensed or controlled by pharmacy department).

0 = No policy, or a policy that does not substantially limit the use of samples (e.g., samples limited for formulary items or samples not for personal use.

**12. Enforcement**

A. Is it clear that there is a party responsible for general oversight to ensure compliance?  (Y/N)

Note for ther coders:  Here we need to check whether the policy says that there is a Committee dedicated to the oversight of the whole policy. (e.g., an Ethics Committee or the Deans Office that check that people are compliant with the policy and that there are no breaches).

B. Is it clear there are sanctions for noncompliance?  (Y/N)
