# Supplementary material for: Content and strength of conflict of interest policies at Scandinavian medical schools: a cross sectional study
Source: BMC Med Educ. 2022 Nov 26;22:812. doi: 10.1186/s12909-022-03881-y (PMC9701355; doi:10.1186/s12909-022-03881-y)
Supplement: Supplementary file 5 — Additional file 5: Supplementary File 5. List of included policies. [file 12909_2022_3881_MOESM5_ESM.docx]

**Supplementary File 5. List of included policies**

| **University** | **Name of policy (with free English translation)** | **University-wide (U) or Medical School policy (S)** | **Date of adoption or most recent review** |
| --- | --- | --- | --- |
| **DENMARK** | | | |
| **Aarhus University*** | Gaver (Gifts) | U | 23/11/2020 |
|  | Bibeskæftigelse (Policy regarding other paid occupation) | U | 23/11/2020 |
|  | Publicering og forfatterskab (Publishing and authorship) | S | 17/12/2020 |
|  | Collaboration agreements with external partners | S | 17/12/2020 |
|  | Arrangementer i Universitetets lokaler (Events on Campus: renting to an external party) | U | 21/12/2020 |
|  | Sponsorpolitik (Guidelines on sponsorship) | U | 01/11/2007 |
| **Aalborg University** | Bibeskæftigelse (Policy regarding other paid occupation) | U | 16/11/2020 |
|  | Gaver og andre fordele (Guideline on gifts) | U | 02/07/2020 |
| **University of Copenhagen** | Københavns Universitetets kodeks for forfatterskaber (Code for Authorship) | U | 22/02/2017 |
|  | Personalepolitisk håndbog (Personnel policy) | U | 24/06/2009 |
| **University of Southern Denmark** | Gaver fra leverandør - bestikkelse (Gifts from suppliers- bribery) | U | 30/07/2020 |
|  | Bibeskæftigelse (Policy regarding other paid occupation) | U | 01/01/2016 |
|  | Sponsorpolitik (Guidelines on sponsorship) | U | 06/03/2012 |
| **NORWAY** |  |  |  |
| **University of Bergen** | Regler om gaver og andre fordeler for ansatte ved UIB (Rules for gifts and other benefits for employees at UIB) | U | 14/02/2008 |
|  | Prinsipper for sidegjøremål for ansatte ved UIB (Principles for side activities for employees at UIB) | U | 25/10/2012 |
|  | 10 etiske regler for UIB (10 ethical rules for UIB) | U | 26/10/2006 |
|  | Regler for bruk av universistetets eiendommer til informasjonsspredning (Rules for the use of the university’s properties for information dissemination) | U | 01/07/2008 |
| **Norwegian University of Science and Technology (NTNU)** | Gave eller bidragsprosjekt (Gift or grant project) | U | 08/10/2019 (latest change) |
|  | Sidegjøremål (Sidejob) | U | 19/11/2020 |
|  | Ansattes forhold til medisinsk industri og næringsliv (Employees relationship with the medical industry and business) | U | 13/11/2019 |
|  | Gaver og fordeler (Gifts and benefits) | U | 05/07/2019 |
|  | Co-authorship | U | 03/06/2020 |
|  | Saksbehandling av sidegjøremål (Case processing of side tasks) | U | 17/04/2018 |
|  | Etikk ved bestilling (Ethics when ordering) | U | 05/06/2018 |
|  | Etiske retningslinjer ved ansatte ved NTNU (Ethical guidelines for employees at NTNU) | U | 26/01/2017 |
| **University of Oslo**** | Retningslinjer for universitetet i Oslo som gavemottager (Guidelines for the university of Oslo as a gift recipient) | U | 01/01/2019 |
|  | Veiledning til reglement for sidegjørempl og eierinteresser ved UIO (Guide to regulations for side activities and ownership interests at UIO) | U | 18/11/2020 |
|  | Interne retningslinjer for forvaltning av bidras og oppdragsfinansiert aktivitet (BOA) ved universitet i Oslo (Internal guidelines for the management of grants and contact finances activity at the University of Oslo) | U | 14/09/2020 |
|  | Universitetets retningslinjer for god vitenskapelig praksis | U | 21/06/2007 |
|  | Veiledning til reglement for sidegjøremål og eierinteresser ved UiO (Guide to the regulations for sideline jobs and owner interests at UIO) | U | 24/08/2020 |
| **University of Tromsø***** | Forfatteradresse og kreditering (Attribution of authorship) This is a webpage and contains a link to an external policy cited in the row below. | U | not stated (it is a webpage) |
|  | Retningslinjer for sidegjøremål (Guideline for side tasks) | U | 14/09/2006 |
| **SWEDEN** | | | |
| **Gothenburg/Sahlgrenska Academy** | Beslut om riktlinjer för medförfattarskap inom Sahlgrenska Akademin (Guidelines on authorship) | U | 12/11/2009 |
|  | Policy avseende mutor och bestickning (Policy regarding bribery) | U | 09/11/2009 |
|  | University of Gothenburg policy for sponsorship | U | 08/12/2014 |
|  | Regler för representation och gåvor (Rules on honoraria and gifts) | U | 23/05/2019 |
|  | Faculty specific rules and regulations for third-cycle studies | S | 13/06/2018 |
|  | Regler om bisysslor och rapportering för anställda vid Göteborgs Universitet (Rules concerning secondary employment) | U | 07/11/2018 |
| **Linkoping University** | Attestordning (Certification order at Linkoping University) | U | 08/04/2020 |
|  | Redovisning bisysslor (Rules regarding disclosure of secondary employment) | U | 24/09/2018 |
|  | Riktlinjer för hantering av avvikelser från god forskningsssed (Instructions concerning violations of good research practice) | U | 02/03/2020 |
|  | Beslut om registrering av avtalad extern finansiering (Registration of external financing related to Linkoping University) | U | 16/11/2016 |
|  | Riktlinjer bisysslor (Rules regarding secondary employment) | U | 24/09/2018 |
|  | Upphandlingspolicy (Procurement policy) | U | 13/02/2019 |
|  | Vägledning om mutor (Guidelines on bribery) | U | 24/05/2017 |
|  | Regler för utbilning på forskarnivå vid Linköpings Universitets Medicinska Fakultet (Rules regarding doctoral studies) | S | 04/06/2019 |
| **Lund University** | Föreskrift bisysslor (Regulations on secondary employment) | U | 10/01/2019 |
|  | Föreskrifter representation (Regulation on financial representation) | U | 21/11/2019 |
|  | Jäv inom forskarutbildning (Conflicts of interest in doctoral education) | S | 29/04/2020 |
|  | Handläggningsordning gällande universitetsgemensam finansiering av forskningsinfrastruktur vid Lunds universitet (Rules regarding university funding of research infrastructure) | U | 14/03/2019 |
|  | Mutor (Webpage regarding rules on bribery) | U | 14/10/2020 |
|  | Föreskrifter om inköp och attest (Regulations regarding procurement) | U | 03/09/2020 |
| **Orebro University** | Riktlinjer representation och gåvor (Guidelines on representation, financial benefits and gifts) | U | 29/10/2019 |
|  | Riktlinjer bisysslor (Rules regarding secondary employment) | U | 21/12/2010 |
|  | Hantering av donationer (Guidelines on handling donations) | U | 25/06/2013 |
|  | Mutor och bestickning (Guidelines on bribery for university employees) | U | 21/12/2010 |
|  | Riktlinjer för sponsring (Guidelines on sponsorship) | U | 01/02/2006 |
|  | Inköps- och upphandlings-policy (Procurement policy) | U | 11/04/2017 |
|  | Riktlinjer för föredragning av avtal (Guidelines on signing financial agreements) | U | 17/09/2013 |
| **Stockholm Karolinska Institute** | Rules governing conflicts of interest | U | 26/04/2006 |
|  | Regler och riktlinjer för att motverka mutbrott och annan otillbörlig påverkan (Rules to prevent bribery) | U | 01/06/2009 |
|  | Bisysslor (Guidelines on secondary employment) | U | 03/09/2020 |
|  | Regler för hantering av bisysslor (Directions on secondary employment) | U | 01/12/2017 |
|  | Tillämpliga bestämmelser om bisysslor (Applicable decisions on secondary employment) | U | 13/09/2016 |
|  | Anvisning för handläggning av bisysslor (On handling secondary employment) | U | 13/09/2016 |
|  | Regler om donationer och sponsring (Rules on donations and sponsorships) | U | 01/01/2019 |
|  | Anvisningar för mottagande av donationer (Regulations on accepting donations) | U | 01/01/2019 |
| **Umea University** | Guidelines for authorship in scientific publications | U | 17/06/2020 |
|  | Bisysslor (Information regarding secondary employment) | U | 17/12/2013 |
|  | Regler och rutiner för medfinansiering av universitetsgemensamma kostnader (Rules and routines concerning financing of expenses within the university) | U | 30/10/2018 |
|  | Policy för mottagande av donationer (Policy on accepting donations) | U | 09/06/2009 |
|  | Sponspringspolicy (Sponsporship policy) | U | 05/02/2013 |
|  | Policy mot tagande och givande av muta (Policy regarding bribery) | U | 30/10/2012 |
| **Uppsala University** | Riktlinjer för uppdragsforskning (Guidelines on contract research) | U | 18/06/2013 |
|  | Rules governing conflicts of interests within the University's area of activity | U | 11/10/2018 |
|  | Riktlinjer sponsoring (Guidelines on sponsorship) | U | 23/06/2009 |
|  | Guidelines on bribery | U | 19/08/2014 |

*For Aarhus University, we found a webpage about collaboration agreements with external partners in which “The Danish Health Act” was referred to. Since the Danish Health Act is an external document, it does not contribute to the final count of policies for Aarhus University.

**For the University of Oslo, we found a referral to “Anbefalinger om kriterier for akademisk forfatterskap” (Recommendations for the Conduct, Reporting, Editing, and Publication of Scholarly Work in Medical Journals by ICMJE (2018) (Vancouver-reglene)). Universitetet i Oslo legger disse anbefalingene til grunn for vitenskapelig forfatterskap. Since the ICMJE guidelines are an external document, they do not contribute to the final count of policies for the University of Oslo.

*** For the University of Tromsø, we assessed this external document whose link was present on one of the University webpages: “Norsk publiseringsindikator (NPI)” (Norwegian publishing indicator). This webpage contains a link to the document “Veiledende retningslinjer for kreditering av vitenskapelige publikasjoner til institusjoner” (Guidelines for attribution of scientific publication). Since it is an external document, it does not contribute to the final count of policies for the University of Tromsø.
